# The Role of the Pathologist in the Next-Generation Era of Tumor Molecular Characterization

**DOI:** 10.3390/diagnostics11020339

**Published:** 2021-02-18

**Authors:** Valentina Angerilli, Francesca Galuppini, Fabio Pagni, Nicola Fusco, Umberto Malapelle, Matteo Fassan

**Affiliations:** 1 Department of Medicine (DIMED), Surgical Pathology Unit, University of Padua, 35121 Padua, Italy; valentina.angerilli@gmail.com (V.A.); francesca.galuppini@unipd.it (F.G.); 2 Department of Medicine and Surgery, Pathology, San Gerardo Hospital, University of Milano-Bicocca, 20900 Monza, Italy; fabio.pagni@unimib.it; 3 Division of Pathology, IEO, European Institute of Oncology IRCCS, 20122 Milan, Italy; nicola.fusco@unimi.it; 4 Department of Oncology and Hemato-Oncology, University of Milan, 20122 Milan, Italy; 5 Department of Public Health, University of Naples Federico II, 80138 Naples, Italy; umbertomalapelle@gmail.com

**Keywords:** molecular pathology, next generation technologies, predictive biomarkers, oncology

## Abstract

Current pathology practice is being shaped by the increasing complexity of modern medicine, in particular of precision oncology, and major technological advances. In the “next-generation technologies era”, the pathologist has become the person responsible for the integration and interpretation of morphologic and molecular information and for the delivery of critical answers to diagnostic, prognostic and predictive queries, acquiring a prominent position in the molecular tumor boards.

## 1. Introduction

In 1953, Crick and Watson’s discovery of the DNA double-helix [1] set a milestone in the history of medicine, deeply transforming the way diseases were viewed. Within the framework of this Kuhnian paradigm shift, the competition of the Human Genome Project a few decades later set the stage for the modern era of precision medicine. As the advent of the microscope led to the engendering of tissue and cellular pathology becoming a thing of the past, the recent introduction of genomic high-throughput technologies in clinical practice is leading the way to a “next-generation” molecularly driven era of pathology.

This “molecular revolution” is providing pathologists with the unique opportunity to gain a novel pivotal role in the therapeutic decision-making process and to be the main actors of the translation of biomarkers discovery into clinical application. In order to take the lead in the genomic transition, pathologists must be equipped with the ability to interpret molecular data and exploit molecular technologies and also willing to expand their horizons to other scientific disciplines, such as bioinformatics and artificial intelligence. However, while embracing the future, molecular pathologists must not underestimate the value of traditional histomorphology in order to provide a comprehensive morpho-molecular diagnosis. Moreover, the adoption of cutting-edge technologies is not without challenges. Many preanalytical and analytical issues should be addressed in order to efficiently integrate molecular profiling in the pathology workflow.

## 2. From the Molecular Alteration to the Targeted Therapy: The Pathologist’s Role

Comprehensive molecular investigations of carcinogenic processes have led to the use of effective molecular targeted agents in solid tumors, thus revolutionizing the current therapeutic strategies in oncology [2]. However, the development of a new generation of drugs targeting specific genes and molecular pathways must be coupled with biomarkers that predict the individual patient’s response to those drugs [3].

As a paradigmatic example, the status of *EGFR*, *ALK*, *ROS1* genes (and more recently that of *MET*, *KRAS*, *ERBB2*, *RET* and *BRAF*) and the expression of PD-L1 should be assessed in patients with lung adenocarcinoma [4]. Similarly, colorectal cancer patients being considered for anti-epidermal growth factor receptor (anti-EFGR) therapy should undergo “extended” *RAS* mutational testing [5]. Moreover, a universal screening approach consisting of defective mismatch repair and microsatellite instability (dMMR/MSI) testing should be performed in all colorectal cancer patients, in order to identify both mismatch repair (MMR) gene mutation carriers and, therefore, Lynch syndrome families [6] and/or patients that can benefit of immunotherapy regimens [7,8]. As for breast cancer, the traditional histopathologic classification has been substituted by a molecular classification, based on the assessment of the proliferation index MIB1, estrogen/progesterone receptor status and *HER2* amplification [9], which have been expanded by *PIK3CA* mutational status in estrogen-positive HER2-negative tumors. More examples of predictive molecular biomarkers routinely tested in clinical settings include the following: *KIT/PDGFRA* in gastrointestinal stromal tumors [10], *BRAF/NRAS/KIT* in advanced melanoma [11,12], *IDH1*/1p/19q codeletion/*MGMT* promoter methylation in brain neoplasms [13]. The main genomic alterations implemented into the clinical management of oncological patients are summarized in Table 1.

Precision Oncology is defined as “the use of therapeutics that are expected to confer benefit to a subset of patients whose cancer displays specific molecular or cellular features (most commonly genomic changes and changes in gene or protein expression patterns)” [16]. Hence, the “molecular revolution” has deeply transformed cancer care, reevaluating the role of the pathologic diagnoses as the backbone of the therapeutic decision-making process [17]. In daily clinical practice, modern pathologists provide an integrated interpretation of the morphologic, molecular and clinical features that drive the therapeutic decisions [18].

The field of precision oncology is rapidly expanding and the number of druggable tumor-specific molecular aberrations has grown substantially in the past decade, with a significant survival benefit in several cancer types [19,20]. For instance, the use of checkpoint-inhibitors immunotherapies has recently demonstrated great efficacy and has been approved for the treatment of many solid tumors [21]. Although, only a subset of patients benefits from these therapies, paving the way for the identification of new biomarkers to stratify patient’s response, such as: immunohistochemical assessment of the programmed death ligand-1 (PD-L1), dMMR/MSI and the tumor mutation burden (TMB) [22,23].

In this fascinating scenario, the discovery, validation and clinical application of novel biomarkers have become a pillar of medical research, pinpointing the need for pathologists to be involved in the translation of biomarker discovery into clinical applications [24].

## 3. The Next-Generation Era of Molecular Diagnostics: Genomics and Beyond

The inclusion of tumor genotyping in the therapeutic decision-making establishes a central role to molecular pathology, which represents the interface between biomarker research and molecular diagnostics. With the advances in molecular diagnostic technologies, highly selective single-gene testing has been outdated by next-generation sequencing (NGS) and other multiplexed platforms in the diagnostic routine [25].

NGS is able to detect a broad spectrum of genetic variations, such as base substitutions, insertions or deletions, copy number alterations, gene rearrangements, gene expression alterations and epigenomics variations [26]. NGS assay can be used to investigate the mutational status of a specific set of genes (targeted panels), to sequence the coding regions of the genome (whole-exome sequencing [WES]) or to sequence the entire genome, including the intronic regions (whole-genome sequencing [WGS]).

At present, targeted panels find large application in clinical settings because they target genes of clinical significance, have greater sensitivity, faster turnaround time and lower cost. On the contrary, despite having a high genomic resolution, WES and WGS are currently being utilized solely for research purposes [27,28]. The major available techniques in molecular diagnostics are summarized in Table 2.

Tumor mutation burden (TMB), defined as the mutation frequency in the tumor genome, has emerged as a promising predictive biomarker of the response to immune checkpoint inhibitors in several prospective trials [29]. The gold standard method to assess TMB for research purposes is WES; however, due to the high cost and lengthy turnaround time of this technique, an alternative approach is the indirect determination of TMB by evaluation of the mutational status of a defined gene panel by NGS [21,23]. Nowadays, from the technical point of view, this is possible in routine settings and also, when scant cellularity samples were the only available biospecimen [30].

The value of performing comprehensive genomic sequencing is exhibited by the recent publication of an integrative analysis of 2658 whole-cancer genomes and their matching normal tissues across 38 tumor types from the Pan-Cancer Analysis of Whole Genomes (PCAWG) Consortium of the International Cancer Genome Consortium (ICGC) and The Cancer Genome Atlas (TCGA) [31].

Genomic profiling focuses on finding discreet driver mutations targeted by therapeutic agents; however, alterations in oncogenes or tumor-suppressor genes do not necessarily predict the activation or inactivation of the corresponding molecular pathway. With transcriptomics being most linked to tumor biology and clinical phenotype, at present, the best way to enrich genomic data is to integrate it with gene-expression analysis data from RNA sequencing [32]. Thus, transcriptomic-based signatures are becoming widely accepted as a relevant source for disease stratification. For example, gene-expression data enriched with additional molecular features have been used to classify colorectal cancer into four consensus molecular subtypes (CMS), which have been found to be independent prognostic factors of survival and relapse [33,34].

The field of precision oncology is currently moving towards a multi-omics approach. Only a comprehensive and integrated analysis of genomic, transcriptomic, epigenomic, metabolomic and proteomic data will be able to unravel the complex mechanisms guiding cancer development and progression. Hence, biomarker research is currently shifting from a “one-gene, one-drug” and “multi-gene, multi-drug” paradigm to a “multi-molecular, multi-drug” perspective [35].

However, generating multi-omics data is expensive, time-consuming and relies on the availability of suitable biopsy material, raising many preanalytical issues. Moreover, advances in computational approaches and bioinformatic pipelines are needed to allow a better stratification of patients’ cohorts into subpopulations able to respond to a given therapy [36].

Although extensive research is still necessary to translate “integromics” in cancer care, a great achievement in precision medicine could derive from a pan-cancer analysis of multi-omics profiles on a genome-wide scale, in order to identify shared actionable targets at a multilayer level across different cancer types [37].

## 4. The Value of an Integrative Morpho-Molecular Approach

As personalized medicine is revolutionizing the way cancer is diagnosed, characterized and treated, it was predicted that molecular biology would replace traditional histopathology. On the contrary, morphology has not disappeared, rather, it has gained even more importance not only for the diagnosis of disease but also to provide adequate and efficient molecular testing [18]. Hence, high-quality microscopy is necessary for high-quality molecular diagnostics.

Morphological evaluation should be performed prior to molecular analysis in order to: (i) confirm the histological subtype of the lesion; (ii) verify that the sample is representative of the disease (i.e., excessive necrotic tissue, inflammation, desmoplastic reaction, mucoid component); (iii) select the area with the highest ratio of malignant to non-malignant cells; (iv) choose the most efficient and cost-effective molecular analysis; (v) identify the presence of artifacts due to preanalytical errors; (vi) evaluate intratumor heterogeneity that may introduce molecular biases [17,24].

Due to the increasing complexity of the molecular pathology landscape, when choosing the proper test and platform for a specific sample, pathologists should proceed according to published molecular testing guidelines [5,38,39], in order to obtain the most accurate and clinically relevant information.

Molecular data may also add diagnostic accuracy in cases of difficult interpretation. For example, the detection of a *BRAF* mutation in an indeterminate thyroid aspiration is highly suggestive of malignancy [40]. Moreover, a comprehensive mutational panel of the most common mutations in papillary and follicular thyroid carcinomas and a gene expression panel have been validated to identify malignancy in cytology samples [41]. In the context of soft tissue pathology, molecular characterization can help overcome the morphologic overlap between subtypes of sarcomas, support diagnosis of tumors arising in non-canonical anatomic locations and distinguish true sarcomas from benignant mimics [42].

On the other hand, molecular information must always be contextualized within an accurate histopathologic evaluation. Indeed, a specific genetic aberration is not a diagnosis itself, but solely a part of one. For example, a mutation in *BRAF* has different prognostic and therapeutic implications across different cancer types. While in melanoma, it is an actionable target for Vemurafenib [43], *BRAF*-mutated colorectal cancer is responsive to a regimen involving the combination of encorafenib, cetuximab, and binimetinib [44].

It may appear that phenotype and genotype are independent; however, the morphological appearance of a lesion under the microscope is an epiphenomenon of its molecular make-up [45]. In fact, a consistency between the histomorphological classification of tumors and a comprehensive molecular profiling (inclusive of genomic, transcriptomic, epigenomic and proteomic data) has been found among different cancer types [46]. Thus, the pathology report of the next-generation sequencing era is characterized by an increasing complexity, as it contains a plethora of morpho-molecular information with a diagnostic, prognostic and predictive value.

## 5. Preanalytical and Analytical Challenges in Molecular Pathology

The performance of molecular testing relies not only on the quality of the method itself, but also, profoundly, on the quality of the biospecimen analyzed. Suboptimal material implies suboptimal results in molecular profiling.

In the last decade, molecular pathology has been implemented in laboratories originally equipped to perform solely traditional histopathology. Furthermore, many methods currently used in pathology are old and suited for a morphological diagnosis. Thus, controlled and standardized management and processing of the biospecimen in the pre-analytical phase is a prerequisite for a reliable and accurate molecular diagnosis. For example, a delay in the transportation from the operating room to the pathology laboratory can cause a degradation of the nucleic acids [47]; to solve this issue, it is possible to transfer tissue under vacuum conditions [48].

Formalin fixation is the most critical step of the pre-analytical phase. A tissue specimen should be fixated preferably in neutral-buffered formalin for 12–24 h. Indeed, a shorter fixation time may cause enzymatic degradation of the tissue, while a longer fixation time may lead to DNA and RNA degradation [49]. The fixation step is particularly detrimental for mRNA, often compromising the performance of gene-expression assays on formalin-fixed paraffin-embedded (FFPE) samples [50].

Furthermore, when cutting FFPE sections, histopathology technicians should follow a strict protocol to minimize the risk of cross-contamination from other samples, which could jeopardize the results of the molecular analysis [51].

However, formalin-fixation is not the only pre-analytical problem to consider in the molecular analysis of samples. These pre-analytic factors usually belong to three great chapters: (i) surgery, (ii) pathology and processing; (iii) sectioning and storage. During surgery, blood vessels are clamped to control bleeding, which results in tissue ischemia and hypoxia. “Warm” ischemic time is the length of time the tissue remains at body temperature after surgical excision; this will be determined by the type of surgery or sample collection method, the specific organ and local clinical practice. ‘Cold’ ischemic time extends from when the excised tissue is chilled (either solely due to cooling to room temperature or being placed on ice) to fixation. The pathologist has to consider this aspect and many laboratories implement procedures to reduce these times and ensure better conservation of the material. Variables identified in the pathology laboratory and during tissue processing include size of tissue sample, fixation conditions, and tissue processing. Generally, it has been recommended that tissues undergo 24 h fixation to initiate the cross-linking chemical reaction and complete the fixation process; however, the fixation time can be decreased when temperatures above room temperature are used. Special techniques are sometimes required to process difficult tissues such as bone samples: decalcifying reagents typically contain a strong acid (nitric acid or hydrochloric acid), a weak acid (picric, acetic, or formic acid), or a chelating agent (EDTA). Bony samples that have undergone strong, acid-based decalcification are compatible with histologic evaluation but are not suitable for PCR-based sequencing procedures, whereas samples that undergo formic acid- or EDTA-based decalcification procedures take longer to process but are suitable for both morphologic analysis and PCR-based sequencing, including NGS. Lastly, the length of storage of the tissue and storage conditions are important in pre-analytic procedures; FFPE tissue samples may be stored for a long period of time without deterioration in histology, but DNA/RNA extraction yield may decrease with increasing storage time. Moreover, storage condition in terms of humidity should be taken into consideration [17].

Tumor-specific factors such as tumor type (solid, cystic or mucinous), specimen size, cellularity and malignant cell fraction can influence DNA yield and, therefore, DNA input, which is a critical quality control step, in relation with the sensitivity of the assay in use [52]. In biospecimens lacking an adequate tumor component, macro- or micro-dissection can be performed to obtain a relatively rich and pure neoplastic cellular population. The pathologist should examine the stained section and meticulously mark the area to be micro-dissected. Manual dissection is preferred in diagnostics, whereas laser-captured microdissection is generally reserved for research purposes [53,54].

Recent advances have enabled the preparation of high-quality NGS libraries from low DNA input, including degraded samples, allowing the use of NGS platforms on a more routine basis [55]. At present, many custom NGS panels have being approved for clinical use in diagnostic pathology, providing a cost- and time-effective solution to perform a comprehensive molecular characterization [56,57,58,59].

Among the most common analytical cofounders in NGS of FFPE samples there are the following: (i) deamination, of cytosine bases [60], which results in C:G>T:A substitutions during amplification, and (ii) amplicon mispriming, which is the main cause of detection of apparent mutations [61]. Phenomena such as these must be recognized during the bioinformatics analysis to avoid significant diagnostic errors. Once the true variants have been identified over the “background noise”, it is important to know whether they are clinically relevant and represent a true pathogenetic event.

One of the main challenges in the use of NGS in a clinical setting is the lack of standardization of platforms, devices and reagents, as well as downstream pipeline data handling, processing and storage. A coordinated effort from companies and academic institutions should be made in order to draw up international guidelines on the management of an NGS-based diagnostic service [62].

## 6. Liquid Biopsy, Digital Pathology and Organoids: New Tools from Translational Research

### 6.1. Liquid Biopsy

In cancer patients, circulating blood and other biological fluids (i.e., urine) have been shown to contain a variety of tumor-associated components, including circulating tumor cells (CTCs), free nucleic acids (circulating tumor DNA [ctDNA], circulating free RNA [cfRNA], miRNA), extracellular vesicles, proteins and metabolites [63]. While being rapid and minimally invasive, the use of liquid biopsy enables the assessment of biomarkers for a variety of malignancies, finding application in diverse clinical scenarios.

In patients with metastatic disease, detection of ctDNA in blood samples is a valuable instrument to identify therapeutically actionable genomic alterations. Moreover, change in the ctDNA burden during treatment is an early predictor of response to treatment [64].

Longitudinal profiling of liquid biopsy samples enables the early identification and characterization of acquired drug resistance and constitutes a powerful tool to map cancer evolution over time [65]. From a public health perspective, liquid biopsy could find application in early detection or screening for cancer in high-risk populations [66,67].

However, the results obtained from a liquid biopsy must be interpreted with caution. In most cases of solid tumors, it will be impossible to replace tissue biopsies because of the limited diagnostic sensitivity in low-tumor-burden patients, the inability to distinguish free non-tumor cell DNA from tumor cell DNA, the lack of information regarding the histological type and tumor microenvironment and the impossibility to perform immunohistochemistry [68].

In many instances, the data obtained from liquid biopsies should therefore be included in the pathology report, alongside the morphological and molecular information acquired from the tissue biopsies, in order to guide the therapeutic decisions.

### 6.2. Digital Pathology

In routine clinical practice, the histopathological diagnosis is based on visual recognition and semi-quantification of morphological patterns. However, despite the use of standardized guidelines, the histopathologic evaluation has an intrinsic subjective nature and is burdened by interobserver and intraobserver variability. The introduction of high-resolution whole-slide imaging, combined with artificial intelligence (AI) and machine learning approaches, could significantly improve the pathology workflow and diagnostic accuracy [69]. For example, the automated scoring of the immunohistochemical expression and the automated count of hybridization signals have led to a quantitative interpretation of results [70]. Furthermore, AI-based analysis of multiple morphometric features on routine hematoxylin and eosin (H&E)-stained preparations can complement the expertise of pathologists in many tasks, such as (i) the detection and quantification of cells and subcellular structures; (ii) the identification of intratumor heterogeneity; (iii) the detection of benignant and malignant areas and inflammation; (iv) grading of the tissue according to severity of disease [71].

Another fundamental point is the need of cutting-edge technical and informatics skills for the use of software at the base of digital pathology. Especially in the early stages of setting up the system, a deep integration between pathologists and engineers will be required to build a system aimed at providing the necessary image analysis for diagnostics and research. A training of the pathologist in the use of the method will be crucial but also a training of the machine itself to “teach it” how to recognize the morphological variables of interest.

### 6.3. Patients’ Derived Organoids (PDO)

Organoids are self-organizing, three-dimensional structures that are grown from stem cells in vitro and resemble of their in vivo organ of origin. At present, large collections of patient-derived tumors and matching healthy organoids are generated from adult stem cells and subsequently, biobanked [72].

Despite being time- and resource-consuming and having some intrinsic limitations, such as the lack of stroma, vascularization and immune cells, patients’ derived organoids (PDO) have emerged as robust pre-clinical models [73].

Small-scale drug screens on organoids biobanks have yielded promising results, as PDOs seem to be able to recapitulate the patient’s response in the clinic, thus paving the way for the development of high-throughput screening [74]. The use of organoids in precision oncology has, once again, stressed the importance of a comprehensive pathologic evaluation of histological features, immunohistochemical markers and molecular profiling to assess treatment response and acquired drug resistance. The use of organoids in clinical practice will represent a translational challenge for the acquisition of different methodologies that refer to pathological, pharmacological and biological fields.

Advantages and disadvantages of these new techniques are summarized in Table 3.

## 7. Are We Ready for the Molecular Revolution?

**Figure 1:**
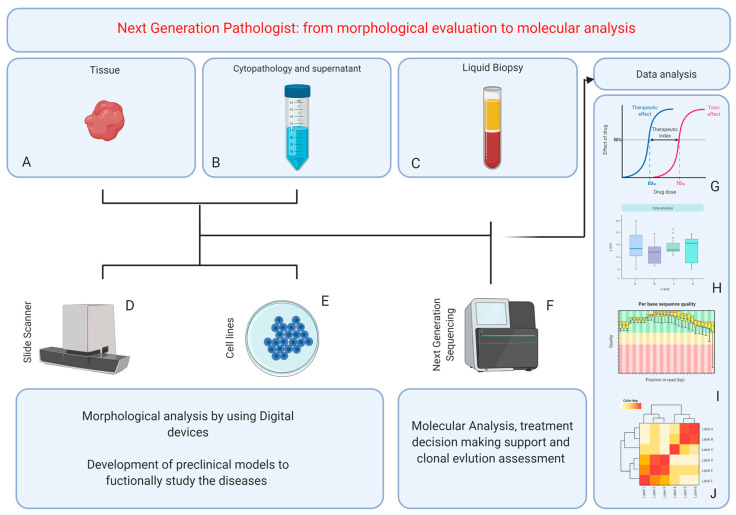
Schematic representation of next-generation pathologist area of expertise, from tissue management and analysis (**A**), including cytological samples and body fluids (**B**,**C**), to fully morpho-molecular characterization (**D**–**F**) of patients. (**G**–**J**): schematic representation of integrative data analysis among drug dose–response (**G**), cell line expression profile (**H**) and next-generation results on DNA (**I**) and RNA (**J**). Credit by Biorender.

As the past decade has seen a rapid decline in the cost of sequencing and genomic platforms, high-throughput sequencing technologies have been implemented in many pathology laboratories [75]. However, only a few “next-generation” pathologists managed to pioneer the “molecular revolution”, merging their unique tissue-based diagnostic skills with the ability to utilize genomic tools and interpret genetic and molecular data (Figure 1). Many traditional pathologists have been reluctant to embrace innovation and often lack a clinically fitting molecular background [76]. Nevertheless, every general pathologist should be able to select the most adequate sample for molecular analysis, understand the performance of genomic tests and integrate morphological and molecular information in the pathology report [17].

To achieve this, molecular diagnostics must be integrated into histopathology training so that pathology residents are equipped with the necessary knowledge to take the lead in the application of genomic technologies. The renewed pathology education curriculum should also include exposure to bioinformatics, to cope with complex pipelines and massive data generated by genomic platforms. Furthermore, pathology residents should be encouraged to take part in translational research projects, to bridge the gap between basic science and clinical applications [77,78].

While every pathologist should be familiar with molecular diagnostics, in each pathology department, there should be subspecialized molecular pathologists with special expertise in high-throughput platforms, genomic medicine and biomarker research, who should also be involved in routine clinical diagnostics. In the past decade, new roles have emerged in the pathology working group, alongside the traditional ones of technicians, biologists and pathologists. The “next-generation” pathology working group now actively involves molecular biologists, laboratory technicians specialized in molecular diagnostics and bioinformaticians [79].

The advent of NGS in the clinic has revolutionized precision oncology; however, interpreting molecular data, matching the patient with the right therapy or clinical trial and addressing primary or acquired drug resistance still remain a challenge. Thus, the increasing complexity of delivering cancer care indicates the need to implement molecular tumor boards (MTBs) widely. MTBs are multidisciplinary forums which should include physicians from different specialties (i.e., medical oncologists, surgeons, radiotherapists, pathologists, clinical geneticists), as well as bioinformaticians, medication–acquisition specialists and clinical trial coordinators [80,81]. MTBs have been recently proven to achieve significantly better clinical outcomes for patients whose physicians followed the MTB discussion recommendations, including longer progression-free survival and overall survival, when compared to patients who received physician’s choice therapy regimen [82].

In the context of MTBs, molecular pathologists, with their unique set of skills, should play a central role in the therapeutic decision-making process, bringing together knowledge in cancer pathology and molecular testing.

## 8. The Key Role of Pathologists in Fighting the COVID-19 Pandemic: A Lesson from the Past

Severe acute respiratory syndrome coronavirus-2 (SARS-CoV-2) marks the unveiling of the third large-scale epidemic related to the coronavirus, after SARS-CoV in 2002 and MERS-CoV in 2012 [83].

The World Health Organization (WHO) declared the SARS-CoV-2 a pandemic on 11 March 2020. The number of confirmed cases as of November 24 is over 58 million with over 1.3 million deaths worldwide [84].

The scientific community has been called on to gain a clear insight on the transmission, physiopathology, clinical features and radiological findings of SARS-CoV-2 infection, in order to obtain reliable diagnostics and optimize clinical management.

Pathologists have worked behind the scenes performing thorough post-mortem examinations, to prove or disprove various postulated clinical events and offer invaluable insights into the pathophysiology of the disease [85]. The understanding of COVID-19-related pathology is rapidly evolving, and further research is needed to help clinicians define better treatment options.

The respiratory system has been the first target of investigation. The most frequently reported pulmonary lesion is the diffuse alveolar damage (DAD), together with hyaline membrane formation and a variable degree of oedema. Nevertheless, these findings cannot be considered highly specific, as they are common in other infectious and non-infectious lung diseases [86,87]. On the contrary, vascular involvement is a distinctive feature of COVID-19, given its tropism for angiotensin-converting enzyme 2 (ACE2). The detection of microvascular injury and microthrombi in lungs and other organs is pathognomonic of a pro-thrombotic state, which has been addressed by clinicians with the introduction of heparin as part as the therapeutic regimen [88].

Although the respiratory tract is the main target of COVID-19, clinical evidences suggest extra-pulmonary involvement. Histopathological lesions related to SARS-CoV-2 infection have been documented in the heart, kidneys, nervous system, skin, testis, liver and gastrointestinal tract [89].

The performance of autopsy has been widely recognized for several decades as a fundamental part of routine pathology practice. In 1761, with the publication of *De Sedibus et Causis Morborum per Anatomen Indagatis* [On the Seats and Causes of Diseases], Giovanni Battista Morgagni placed anatomo-clinical correlations at the heart of modern medicine [90]. The lesion in the organ, revealed by the post-mortem examination, is considered by Morgagni as the fundamental cause of disease and of its origin, progress, and clinical manifestations [91].

However, since the last decades of the twentieth century, the rate of autopsies has decreased significantly, due to increasing confidence in ante-mortem diagnosis, more complex legislations regarding human tissues procedures and also insufficient priority given to autopsies by pathologists themselves, burdened with increasing workloads of surgical resections, biopsies, and cytology [92].

The declining autopsy rate should be a source of concern among the scientific community, given its great potential for the advancement of medical knowledge and improvement of clinical practice. As demonstrated by the COVID-19 experience, the post-mortem examination, together with state-of-the-art molecular diagnostics, plays a central role in the diagnosis and clinical management of newly emerging diseases [93].

## 9. Conclusions

Current pathology practice is being shaped by the increasing complexity of modern medicine and major technological advances [14]. In the “next-generation sequencing era” the pathologist has become the clinician responsible for the integration and interpretation of morphologic and molecular information and for the delivery of critical answers to diagnostic, prognostic and predictive queries, acquiring a prominent position in the personalized medicine scenario, especially in cancer care.

However, traditional organ, tissue and cellular pathology lays the foundation for the development of molecular pathology and, as demonstrated by the on-going COVID-19 pandemic, still provides essential tools to greatly enhance clinical practice.

Current practicing and future pathologists are called on to actively incorporate molecular knowledge into their diagnostic armamentarium and deeply transform laboratory frameworks and pathology educational training programs.

We are facing an epochal evolution of the figure of the pathologist who must necessarily find fertile ground both in laboratories, thanks to economic availability and mental openness towards innovation, and also in the training programs of young pathologists. Studies aimed at new technologies will be a fundamental background for the pathologist–in-training.

Collaboration between different professional figures must acquire more and more importance in the context of the MTB. The first experiences of multidisciplinary teams in the molecular field are demonstrating how an integration between different knowledge in specialized fields such as clinical, molecular and surgical pathology and engineering can lead to an increasingly targeted therapy, to an increasingly personalized follow-up and a more precise cancer risk stratification.

## Figures and Tables

**Table 1:** Main genomic alterations in different tumor types, as assessed within the clinic.

Tumor Types	Genomic Alteration Involved	References
Lung non-squamous non-small cell lung cancer (NSCLC)	*EGFR* (exons 18-21 mutations/deletions and acquired T790M mutation); *ALK* and *ROS1* fusions, *MET* amplifications and mutations; *KRAS* mutations; *ERBB2* mutations and amplifications; *RET* fusions; *BRAF* mutations; *NTRK* fusions; PD-L1 expression	[4]
Colorectal adenocarcinoma	*KRAS*, *NRAS* and *BRAF* mutations; microsatellite instability/defective mismatch repair (MSI/dMMR)	[7]
Breast cancer	*ERBB2* amplifications; *PIK3CA* mutations; ER/PR expression; *BRCA1/BRCA2* germline mutations; PD-L1 expression; Oncotype Dx	[9,14]
GIST	*KIT* and *PDGFRA* mutations	[10]
Melanoma	*BRAF*, *CDKN2A* and *KIT* mutations	[11]
Pancreatic adenocarcinoma	*BRCA1* and *BRCA2* mutations (somatic and germline)	[14]
Brain neoplasms	*IDH1/IDH2* mutations; 1p/19q co-deletion; *MGMT* promoter methylation	[13]
Gastric cancer	MSI/dMMR; *ERBB2* amplifications; PD-L1 expression	[14]
Prostate cancer	*BRCA1* and *BRCA2* mutations (somatic and germline); *ATM* mutations (germline)	[14]
Endometrial cancer	MSI/dMMR; *TP53* mutations; *POLE* mutations	[14]
Ovarian cancer	*BRCA1* and *BRCA2* mutations (somatic and germline); *ATM* mutations; BRiP1 mutations; CHEK2 mutations; PALB2 mutations; RAD51C/RAD51D mutations	[14]
Thyroid cancer	*BRAF* and *RAS* mutations; *hTERT* promoter mutations	[15]

**Table 2:** Major available techniques in molecular diagnostics in the clinic.

**Techniques**	**Advantages**	**Disadvantages**
Sanger sequencing	- Low-cost machinery - Widespread on a large scale in all molecular biology laboratories	- Higher turn-around-time in comparison to NGS technologies - Low sensitivity - Limited information on tumor molecular landscape
MALDI-TOF mass spectrometry sequencing	- Widespread on a large scale in all molecular biology laboratories - Possibility to use specific gene panels - Greater sensitivity than Sanger sequencing	- Lower sensitivity than NGS
Reverse transcriptase (RT)-PCR	- Great sensitivity in detecting fusion genes - Low turn-around-time - Widespread on a large scale in all molecular biology laboratories	- Alteration-specific primers - RNA-based
qRT-PCR	- Great diagnostic sensitivity - Low turn-around-time - Widespread on a large scale in all molecular biology laboratories - Reliable for liquid biopsy analysis	- Alteration-specific primers
Pyrosequencing	- Low-cost machinery - Widespread on a large scale in all molecular biology laboratories - Best performances in studies on methylation	- Limited information on tumor molecular landscape
Digital droplet PCR (ddPCR)	- High diagnostic sensitivity - Reliable for liquid biopsy analysis	- Limited information on tumor molecular landscape
Immunohistochemistry	- Low turnaround-time - Widespread on a large scale in all molecular biology laboratories	- More affected by preanalytical artifacts than molecular pathology diagnostics
Next-generation sequencing (NGS) targeted panels	- Greater sensitivity - Allows lot of targeted fragments to be sequenced in a single run - Faster turnaround time - Lower cost than comprehensive profiling.	- Biostatistical analysis of the results
Whole-genome sequencing (WGS)	- Identifies meaningful mutations even they occur outside of exons - GC-rich gene sequences appear more accurately captured	- Difficult interpretation of genomic data - Relatively expensive
Whole-exome sequencing (WES)	- Cost-effective alternative to WGS - Focuses on the most relevant portion of the genome and facilitates the discovery and validation of common and rare variants	- Unable to interrogate many variants that may be important for controlling gene transcriptional regulation or splicing

**Table 3:** Liquid biopsy, digital pathology and patient’s derived organoid: advantages and disadvantages.

**Techniques**	**Advantages**	**Disadvantages**
Liquid biopsy	- Noninvasive test - Quick result turnarounds	- Low specificity, especially as the list of biomarkers expands with better understanding of the underlying biology of cancers
Digital pathology	- Improves data and analysis quality - Low storage costs - Easier sharing of histological slides	- Adds time and cost to the typical surgical pathology clinical workflow - Expensive machinery - Need for analysis software and skills for their use
Patients’ derived organoids (PDO)	- Effective biological model to test the in vitro effect of drugs	- Time- and resource-consuming - Lack of some cellular and molecular background of the natural tissue
**Techniques**	**Advantages**	**Disadvantages**
Liquid biopsy	- Noninvasive test - Quick result turnarounds	- Low specificity especially as the list of biomarkers expands with better understanding of the underlying biology of cancers
Digital pathology	- Improves data and analysis quality - Low storage costs - Easier sharing of histological slides	- Adds time and cost to the typical surgical pathology clinical workflow - Expensive machinery - Need for analysis software and skills for their use
PDO	- Effective biological model to test the in vitro effect of drugs	- Time- and resource-consuming - Lack of some cellular and molecular background of the natural tissue

## Data Availability

Not applicable.
